# Inhibition of cathepsin K sensitizes oxaliplatin-induced apoptotic cell death by Bax upregulation through OTUB1-mediated p53 stabilization in vitro and in vivo

**DOI:** 10.1038/s41388-021-02088-7

**Published:** 2021-11-16

**Authors:** Seung Un Seo, Seon Min Woo, Shin Kim, Jong-Wook Park, Hyun-Shik Lee, Young-Seuk Bae, Sang Hyun Kim, Seung-Soon Im, Ji Hae Seo, Kyoung-jin Min, Taeg Kyu Kwon

**Affiliations:** 1 Department of Immunology, School of Medicine, Keimyung University, Daegu, 42601 South Korea; 2 School of Life Sciences, BK21 Plus KNU Creative BioResearch Group, College of Natural Sciences, Kyungpook National University, Daegu, 41566 South Korea; 3 Department of Pharmacology, School of Medicine, Kyungpook National University, Daegu, 41944 South Korea; 4 Department of Physiology, School of Medicine, Keimyung University, Daegu, 42601 South Korea; 5 Department of Biochemistry, Keimyung University School of Medicine, Daegu, 42601 South Korea; 6 New Drug Development Center, Daegu-Gyeongbuk Medical Innovation Foundation (DGMIF), Daegu, 41061 South Korea; 7 Center for Forensic Pharmaceutical Science, Keimyung University, Daegu, 42601 South Korea

**Keywords:** Cancer therapeutic resistance, Apoptosis

## Abstract

Cathepsin K is highly expressed in various types of cancers. However, the effect of cathepsin K inhibition in cancer cells is not well characterized. Here, cathepsin K inhibitor (odanacatib; ODN) and knockdown of cathepsin K (siRNA) enhanced oxaliplatin-induced apoptosis in multiple cancer cells through Bax upregulation. Bax knockdown significantly inhibited the combined ODN and oxaliplatin treatment-induced apoptotic cell death. Stabilization of p53 by ODN played a critical role in upregulating Bax expression at the transcriptional level. Casein kinase 2 (CK2)-dependent phosphorylation of OTUB1 at Ser16 played a critical role in ODN- and cathepsin K siRNA-mediated p53 stabilization. Interestingly, ODN-induced p53 and Bax upregulation were modulated by the production of mitochondrial reactive oxygen species (ROS). Mitochondrial ROS scavengers prevented OTUB1-mediated p53 stabilization and Bax upregulation by ODN. These in vitro results were confirmed by in mouse xenograft model, combined treatment with ODN and oxaliplatin significantly reduced tumor size and induced Bax upregulation. Furthermore, human renal clear carcinoma (RCC) tissues revealed a strong correlation between phosphorylation of OTUB1(Ser16) and p53/Bax expression. Our results demonstrate that cathepsin K inhibition enhances oxaliplatin-induced apoptosis by increasing OTUB1 phosphorylation via CK2 activation, thereby promoting p53 stabilization, and hence upregulating Bax.

## Introduction

Cathepsin K is one of the major proteases in the lysosomal cysteine protease family. Cathepsin K has functional roles in multiple physiological processes, such as MHC-II-mediated antigen presentation, bone resorption, and keratinocyte differentiation [1, 2]. Cathepsin K expression is elevated in cancer cells [3]. Cathepsin K acts in cancer progression and invasion by indirectly or directly degrading extracellular matrix proteins [3]. Cathepsin K inhibitors have been proposed for the treatment and prevention of bone cancer and bone metastases [4]. Odanacatib (ODN) is a small-molecule selective cathepsin K inhibitor that prevents binding to its substrates. Combined treatment with SM934 (a novel water-soluble artemisinin analog) and testosterone inhibited proliferation and metastasis of cancers by inhibiting cathepsin K expression, which in turn inhibited Bcl-xL [5]. Recently, ODN has been found to enhance TNF-related apoptosis-inducing ligand (TRAIL) sensitivity via ubiquitin-specific peptidase 27x (USP27x)-mediated Bim upregulation [6]. ODN induces proteasome-dependent degradation of regulatory associated protein of mammalian target of rapamycin (Raptor), followed by production of mitochondrial ROS, which has a critical role in USP27x-mediated Bim stabilization [6]. Furthermore, cathepsin K overexpression promotes cell proliferation, migration, and invasion in non-small cell lung cancer [7], and cathepsin K inhibition prevents the establishment and progression of prostate cancer in bone [8].

Oxaliplatin, a platinum compound, is one of the most heavily utilized chemotherapeutic agents for colorectal cancer [9, 10]. Oxaliplatin exerts its cytotoxic effect chiefly by inducing DNA strand breakage and inhibiting DNA synthesis and repair, ultimately leading to apoptosis. However, the anticancer effect of oxaliplatin is limited by intrinsic or acquired drug resistance. Oxaliplatin has also been administered in combination therapy as an effective strategy for reducing adverse side effects and resistance in various types of cancers. Further study is required to understand oxaliplatin’s mechanisms of cytotoxicity and to identify chemical reagents that improve its anticancer effects. This will lead to more effective anticancer drug treatment strategies.

Therefore, our objectives were to understand how ODN affects oxaliplatin-induced apoptosis and to identify the molecular mechanisms by which combined oxaliplatin and ODN treatment induce apoptosis in human renal carcinoma cells.

## Results

### Cathepsin K inhibition and knockdown enhances oxaliplatin-induced apoptosis

**Fig. 1:**
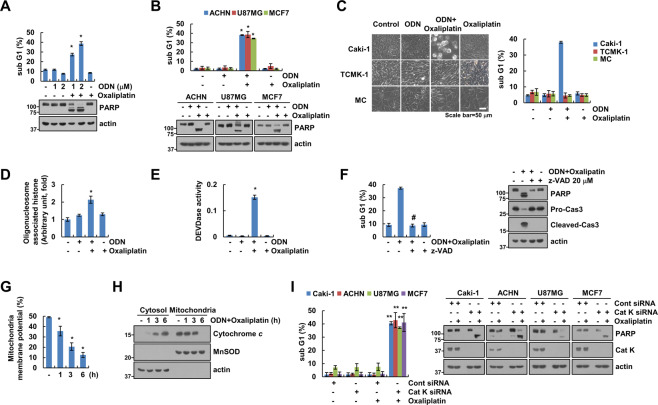
Cathepsin K inhibition sensitizes oxaliplatin-mediated apoptosis. **A–E** Cancer or normal cell lines were treated with a 25 μM oxaliplatin or/and 2 μM odanacatib (ODN) for 24 h. Quantification of DNA fragments was determined using a DNA fragmentation assay kit (**D**). Detection of caspase activity was measured using a DEVDase colorimetric assay kit (**E**). **F** Caki-1 cells were treated with a combination of 2 μM ODN and 25 μM oxaliplatin in the presence or absence of a pan-caspase inhibitor, 20 μM z-VAD-fmk (z-VAD), for 24 h. **G, H** Caki-1 cells were treated with a combination of 2 μM ODN and 25 μM oxaliplatin for the indicated times. Flow cytometry was used to detect fluorescence intensity to measure MMP, using rhodamine 123 fluorescent dye (**G**). Cytochrome *c* release was analyzed by cytoplasmic fraction. MnSOD was used as a mitochondrial fraction marker (**H**). **I** The cancer cell lines were transfected with control siRNA or cathepsin K siRNA and were treated with 25 μM oxaliplatin for 24 h. Apoptosis and protein expression were measured by flow cytometry (**A–C, F**, and **I**) and western blotting (**A**, **B**, **F**, **H** and **I**). Cell morphology was assessed using a microscope; scale bar: 50 µm (**C**). The values in the graphs **A**–**G**, and **I** represent the mean ± SD of three independent experiments. *P < 0.01 compared to the control. ^#^*P* < 0.01 compared to the ODN-plus-oxaliplatin combination. **P < 0.01 compared to the cathepsin K siRNA-transfected cells treated with oxaliplatin.

We examined whether the cathepsin K inhibitor ODN enhanced oxaliplatin-induced apoptosis. ODN alone, or a sub-lethal dose of oxaliplatin (25 µM), did not increase the sub-G1 population or PARP cleavage. In contrast, combined treatment with ODN and oxaliplatin (25 µM) markedly increased the sub-G1 population and induced PARP cleavage in renal carcinoma cells (Caki-1 and ACHN), glioma (U87MG) cells, and breast carcinoma (MCF7) cells (Fig. 1A, B). Combined treatment with ODN and oxaliplatin-induced detachment on plate and cell shrinkage in Caki-1 cells, but not in normal human renal mesangial cells (MC) or TCMK-1 cells (Fig. 1C). Combined treatment also induced cytoplasmic histone-associated DNA fragmentation (Fig. 1D). Furthermore, ODN plus oxaliplatin-induced caspase-3 activation (Fig. 1E), and z-VAD-fmk (z-VAD; a pan-caspase inhibitor) inhibited ODN-plus-oxaliplatin-induced sub-G1 population and PARP cleavage (Fig. 1F). This indicates that combined oxaliplatin and ODN treatment induces caspase-dependent apoptotic cell death.

Loss of mitochondrial membrane potential (MMP) plays a critical role in apoptosis, by releasing cytochrome *c* into the cytoplasm [11]. Therefore, we examined the effect of the combined treatment on MMP. The combined treatment induced a reduction in MMP (Fig. 1G) and an increase in cytosolic cytochrome *c* levels (Fig. 1H). To verify the involvement of cathepsin K inhibition in sensitization to oxaliplatin-induced apoptosis, we examined apoptosis using cathepsin K siRNA. Oxaliplatin alone markedly increased the sub-G1 population and induced PARP cleavage in cathepsin K siRNA transfected all p53 wild-type (WT) cancer cells, including Caki-1, ACHN, U87MG, and MCF7 cells (Fig. 1I). Therefore, our data indicate that cathepsin K inhibition enhanced oxaliplatin-mediated apoptosis in multiple cancer cells.

### Upregulation of Bax expression plays a critical role in combined treatment with a cathepsin K inhibitor and in oxaliplatin-induced apoptosis

**Fig. 2:**
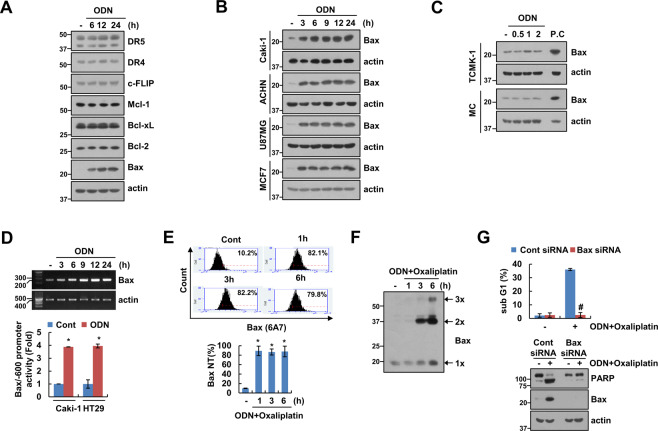
ODN increases Bax expression at the transcriptional level. **A–C** The cancer (**A, B**) and normal cell (**C**) lines were treated with 2 μM ODN for the indicated times. **D** Caki-1 cells were treated with 2 μM ODN for the indicated times. mRNA expression was measured using RT-PCR (upper panel). The cancer cell lines were transiently transfected with a Bax/−600 promoter and incubated with 2 μM ODN for 12 h. The cells were lysed, and promoter activity was measured (lower panel). **E** Caki-1 cells were treated with a combination of 2 μM ODN and 25 μM oxaliplatin for the indicated times. Flow cytometry was used to detect the fluorescence intensity of active Bax, using conformation-specific antibodies. **F** For Bax oligomerization assay, monomers, and oligomers of Bax were quantified by western blotting. **G** Caki-1 cells were transfected with control siRNA or Bax siRNA, and treated with a combination of 2 μM ODN and 25 μM oxaliplatin for 24 h. Protein expression and apoptosis were measured using western blotting (**A–C**, **F**, and **G**) and flow cytometry (**E, G**). The values in the graphs **D**, **E**, and **G** represent the mean ± SD of three independent experiments. *P < 0.01 compared to the control. ^#^*P* < 0.01 compared to the Bax siRNA-transfected cells treated with ODN plus oxaliplatin.

To elucidate the molecular mechanisms leading to apoptosis in ODN-treated cells, we analyzed the regulation of apoptosis-related protein expression. The expression of the apoptosis-related proteins that we studied was not altered by ODN treatment in Caki-1 cells (Fig. 2A). However, ODN significantly upregulated Bax, a proapoptotic protein. Further, ODN-induced Bax upregulation in ACHN, U87MG, and MCF7 cells, but not normal MC and normal TCMK-1 cells (Fig. 2B, C). ODN increased Bax mRNA expression and its promoter activity in cancer cells (Fig. 2D). Since Bax activation plays a critical role in the release of cytochrome *c* from the mitochondria [26], we examined Bax activation using conformation-specific anti-Bax antibodies (6A7) in ODN-plus-oxaliplatin-treated cells. Combined treatment markedly induced Bax activation and Bax oligomerization (Fig. 2E, F). We subsequently investigated the functional importance of Bax. Bax knockdown via siRNA markedly inhibited ODN-plus-oxaliplatin-induced apoptosis and PARP cleavage (Fig. 2G). Therefore, our findings show that upregulation of Bax expression is associated with ODN-plus-oxaliplatin-induced apoptosis.

### P53 upregulation plays a critical role in ODN-induced Bax expression

**Fig. 3:**
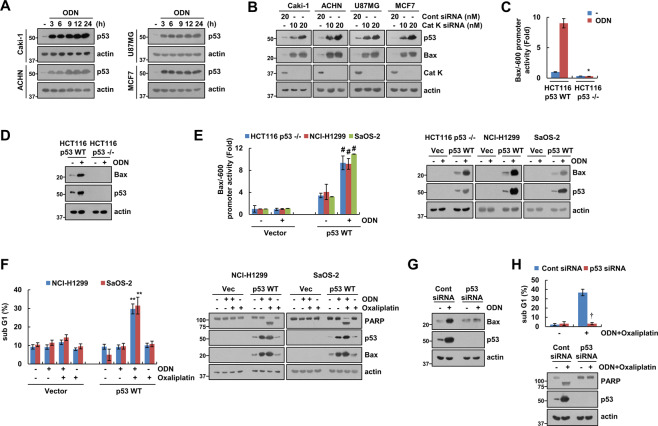
Increased p53 expression contributes to ODN plus oxaliplatin-induced Bax upregulation and apoptosis. **A** The cancer cell lines were treated with 2 μM ODN for the indicated times. **B** Cathepsin K-knockdown cell lines were used. **C, D** HCT116 p53 WT and p53-null cells were transiently transfected with a Bax/−600 promoter and incubated with 2 μM ODN for 12 h. The cells were lysed, and promoter activity and expression levels of Bax and p53 were measured. **E** The cancer cell lines were transiently cotransfected with vector or p53 WT and a Bax/−600 promoter and were treated with/without 2 μM ODN for 12 h, and promoter activity and expression levels of Bax and p53 were measured. **F** The cancer cell lines were transiently cotransfected with vector or p53 WT, and were treated with a combination of 25 μM oxaliplatin in the presence or absence of 2 μM ODN for 24 h. **G, H** Caki-1 cells were transfected with control siRNA or p53 siRNA, and treated with 2 μM ODN (**G**) or a combination of 2 μM ODN and 25 μM oxaliplatin (**H**) for 24 h. Protein expression and apoptosis were measured using western blotting (**A, B, D**, and **E–H**) and flow cytometry (**F** and **H**). The values in the graphs **C, E, F**, and **H** represent the mean ± SD of three independent experiments. *P < 0.01 compared to the ODN in HCT116 p53 WT cells. ^#^*P* < 0.01 compared to the ODN in the vector-transfected cells. **P < 0.01 compared to the ODN plus oxaliplatin in the vector-transfected cells. †*P* < 0.01 compared to the control siRNA-transfected cells treated with ODN plus oxaliplatin.

To identify the molecular mechanism of Bax upregulation, we investigated the expression of p53, a key transcriptional factor of Bax [12]. ODN increased p53 protein expression within 3 h, in all of the p53 WT cancer cell lines that we tested (Fig. 3A). Cathepsin K knockdown via siRNA also upregulated p53 and Bax expression in p53 WT cancer cells (Fig. 3B). Furthermore, ODN did not increase Bax promoter activity and Bax expression in HCT116 p53-null cells (Fig. 3C, D). However, cotransfection with the Bax-promoter construct and p53-expression plasmid increased Bax promoter activity and expression in p53-null cell lines (Fig. 3E). Transfection of p53 markedly increased combined ODN and oxaliplatin-induced apoptosis, PARP cleavage, and Bax upregulation in p53-null NCI-H1299 and SaOS-2 cells, but not in vector-transfected cells (Fig. 3F). In addition, siRNA-mediated p53 knockdown completely blocked ODN-induced Bax expression (Fig. 3G) and abolished ODN-plus-oxaliplatin-induced apoptosis and PARP cleavage in p53 WT Caki-1 cells (Fig. 3H). Taken together, these results indicate that p53 upregulation induced by ODN is involved in Bax upregulation, thereby contributing to the sensitization to oxaliplatin-mediated apoptosis in p53 WT cancer cells.

### ODN induces p53 upregulation via OTUB1 phosphorylation

Next, we investigated the mechanism whereby ODN induces p53 expression. p53 WT Caki-1 cells were treated with or without ODN in the presence of cycloheximide (CHX). Including ODN significantly enhanced p53 stabilization compared with using CHX alone (Fig. S1A). Because p53 stability is mainly regulated by the MDM2 E3 ligase [13], we investigated the effect of ODN on MDM2 expression. ODN did not alter MDM2 expression (Fig. S1B). Furthermore, Nutlin-3 (a small-molecule MDM2 antagonist) and MDM2 siRNA did not affect ODN-mediated p53 and Bax expression (Fig. S1C, D). Therefore, these findings suggest that ODN-induced p53 stabilization is not associated with MDM2.

**Fig. 4:**
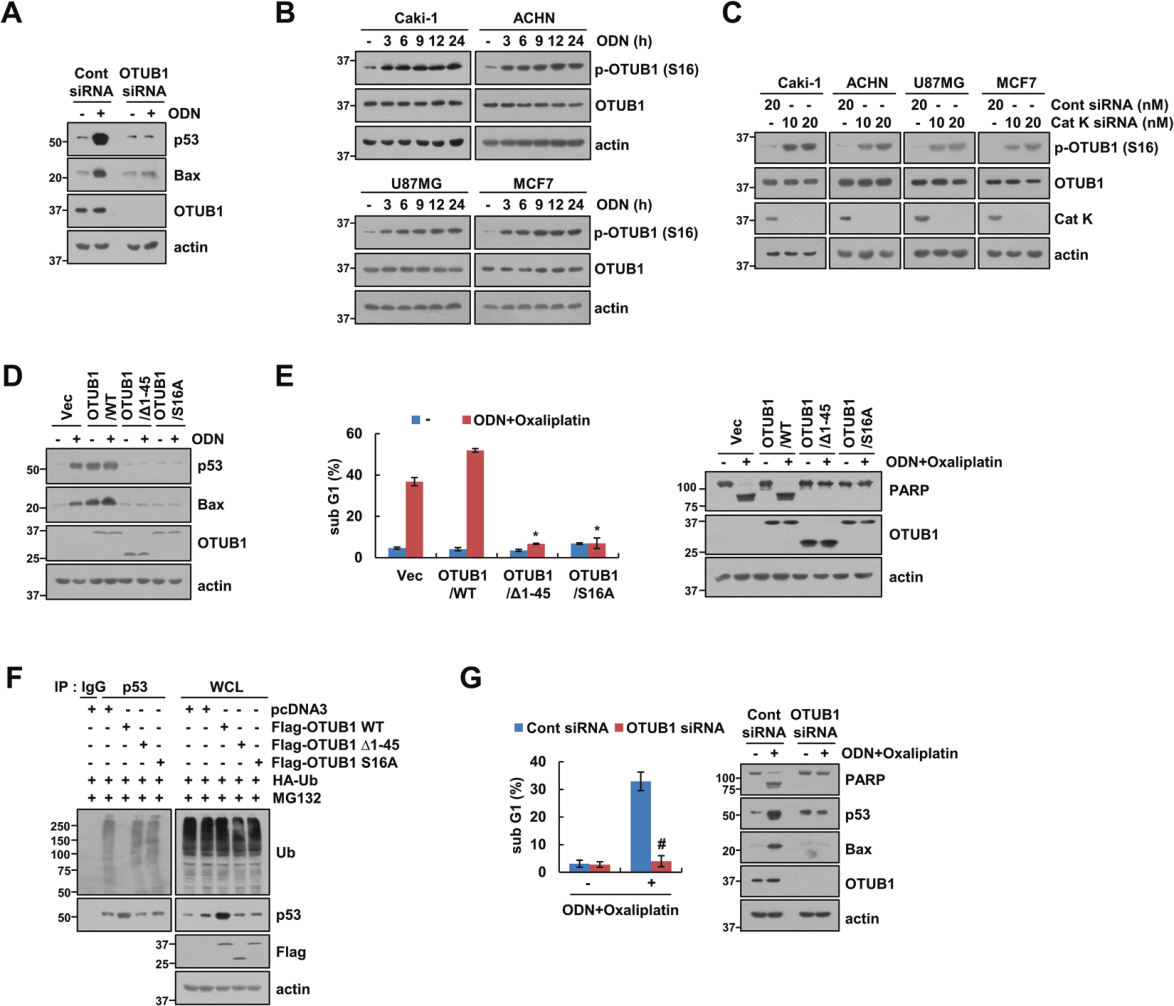
ODN upregulates p53 expression via OTUB1 phosphorylation at Ser16. **A** Caki-1 cells were transfected with control siRNA or OTUB1 siRNA, and treated with 2 μM ODN for 24 h. **B** The cancer cell lines were treated with 2 μM ODN for the indicated times. **C** The cancer cell lines were transfected with control siRNA or cathepsin K siRNA. **D, E** Caki-1 cells were transiently transfected with the vector, OTUB1 WT, OTUB1 Δ1-45, or OTUB1 S16A, and treated with 2 μM ODN (**D**) or a combination of 2 μM ODN and 25 μM oxaliplatin (**E**) for 24 h. **F** To analyze p53 ubiquitination, Caki-1 cells were cotransfected with the vector, OTUB1 WT, OTUB1 Δ1-45, or OTUB1 S16A and HA-ubiquitin (HA-Ub), and were treated with 0.5 μM MG132 for 12 h. Immunoprecipitation was performed using an anti-p53 antibody. **G** Caki-1 cells were transfected with control siRNA or OTUB1 siRNA, and treated with a combination of 2 μM ODN and 25 μM oxaliplatin for 24 h. Protein expression and apoptosis were measured using western blotting (**A–G**) and flow cytometry (**E**, **G**). The values in the graphs **E**, **G** represent the mean ± SD of three independent experiments. *P < 0.01 compared to the vector-transfected cells treated with ODN plus oxaliplatin. ^#^*P* < 0.01 compared to the control siRNA-transfected cells treated with ODN plus oxaliplatin.

Deubiquitination plays a role in regulating p53 stability [14]. Specifically, ovarian tumor domain-containing ubiquitin (Ub) aldehyde-binding protein 1 (OTUB1) modulates p53 stability [14]. Therefore, we investigated whether OTUB1 modulates ODN-induced p53 stabilization. Downregulation of OTUB1 by siRNA markedly inhibited ODN-induced p53 stabilization and Bax expression (Fig. 4A). However, ODN did not affect OTUB1 protein expression in Caki-1 cells (Fig. 4A). Interestingly, ODN and cathepsin K siRNA induced phosphorylation of the Ser residue 16 (S16) of OTUB1 in p53 WT cancer cell lines (Fig. 4B, C). Therefore, we investigated the effects of the OTUB1 WT, mutant (S16A), and N-terminal deletion mutant (Δ1-45) on ODN-induced p53 stabilization and Bax upregulation. The OTUB1 mutant (S16A) and N-terminal deletion mutant inhibited ODN-induced p53 stabilization and Bax upregulation in p53 WT Caki-1 cells (Fig. 4D). Also, combined treatment ODN plus oxaliplatin did not induce apoptosis in OTUB1 mutant (S16A)- and N-terminal deletion mutant-transfected cells (Fig. 4E). Furthermore, although OTUB1 dramatically induced p53 deubiquitination, its mutant (S16A) and deletion mutant did not have this effect (Fig. 4F). In addition, OTUB1 knockdown inhibited ODN-plus-oxaliplatin-induced apoptosis and PARP cleavage in p53 WT Caki-1 cells (Fig. 4G). These findings indicate that cathepsin K inhibition increases p53 stabilization via phosphorylation of OTUB1 in p53 WT cancer cell lines.

### Casein kinase 2 (CK2) phosphorylates OTUB1

**Fig. 5:**
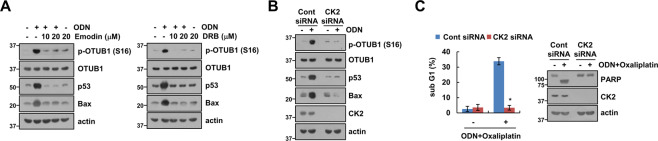
CK2 is involved in OTUB1 phosphorylation-dependent p53 stabilization by ODN. **A** Caki-1 cells were pretreated with CK2 inhibitors (emodin and DRB) for 30 min, then treated with 2 μM ODN for 24 h. **B, C** Caki-1 cells were transfected with control siRNA or CK2 siRNA and treated with 2 μM ODN (**B**) or a combination of 2 μM ODN and 25 μM oxaliplatin (**C**) for 24 h. Protein expression and apoptosis were measured using western blotting (**A–C**) and flow cytometry (**C**). The values in the graphs **C** represent the mean ± SD of three independent experiments. *P < 0.01 compared to the control siRNA-transfected cells treated with ODN plus oxaliplatin.

We investigated how ODN induces phosphorylation of OTUB1 at Ser16. Since CK2 is a critical kinase in Ser16 phosphorylation in OTUB1 [15], we examined the role of CK2 in this process. The pharmacological CK2 inhibitors [emodin and 5,6-dichlorobenzimidazole 1-beta-D-ribofuranoside (DRB)] significantly inhibited ODN-induced OTUB1 phosphorylation, p53 stabilization, and Bax expression (Fig. 5A). Genetic ablation of CK2 by siRNA revealed similar results (Fig. 5B). Furthermore, CK2 knockdown by siRNA inhibited ODN-plus-oxaliplatin-induced apoptosis and PARP cleavage (Fig. 5C). These findings indicate that CK2 contributes to p53 stabilization via OTUB1 phosphorylation in ODN-treated Caki-1 cells.

### Mitochondrial ROS production is critical for ODN-mediated p53 stabilization

**Fig. 6:**
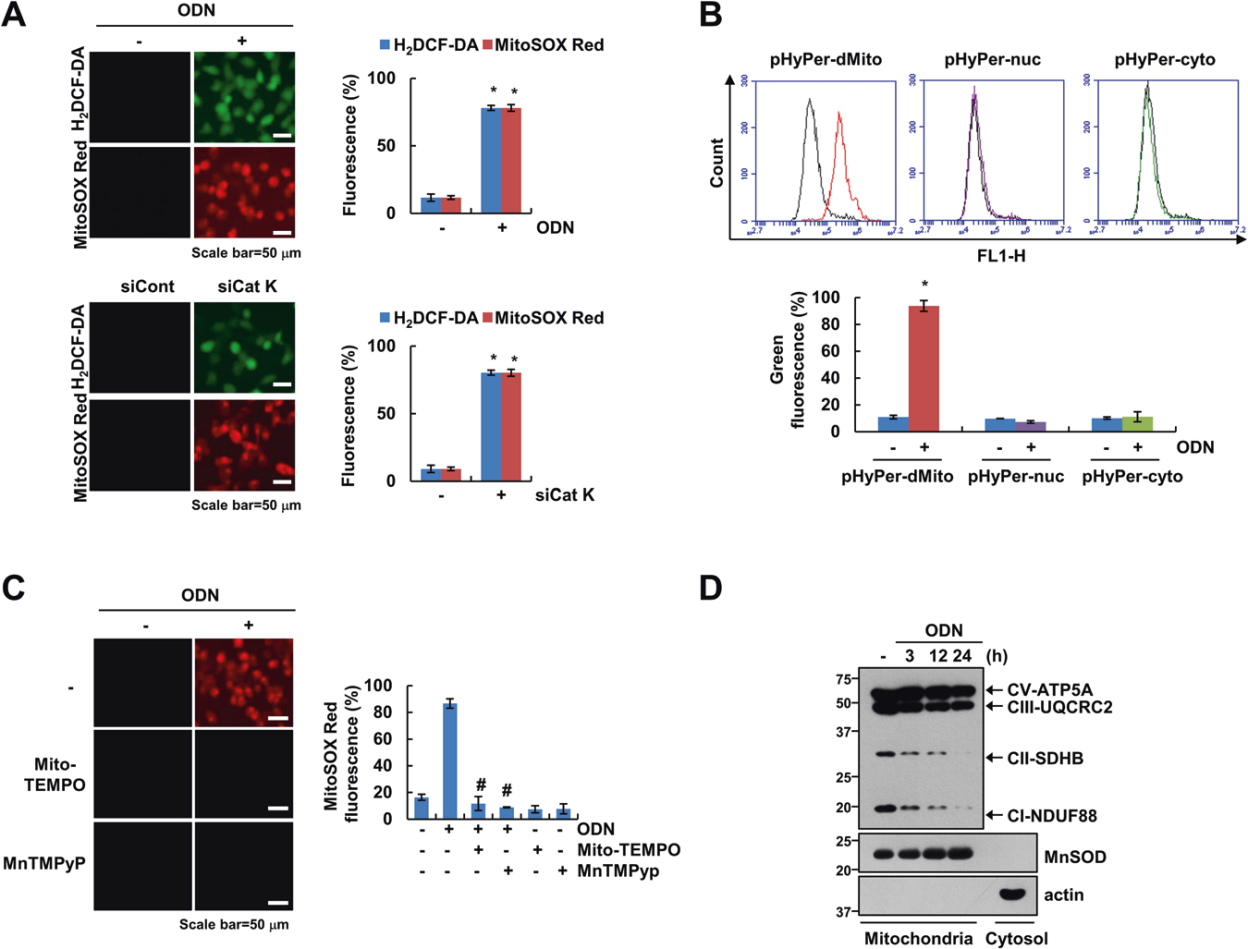
ODN induces mitochondrial ROS generation. **A** Caki-1 cells were treated with/without 2 μM ODN or transfected with control siRNA or Cat K siRNA, and the cells were loaded with H_2_DCF-DA (green) and Mitosox Red (red) fluorescent dye. **B** Caki-1 cells were transfected with pHyper-Mito, pHyper-nuc, or pHyper-cyto vector. After 24 h, cells were treated ODN for 3 h and then detected of green fluorescence. **C** Caki-1 cells were pretreated with mitochondrial ROS scavengers (Mito-TEMPO and MnTMPyp) for 30 min, then treated with 2 μM ODN for 3 h. The H_2_DCF-DA and Mitosox Red fluorescence intensity was detected by a fluorescence microscope and flow cytometry. **D** Caki-1 cells were treated with 2 μM ODN for the indicated times, and OXPHOS proteins of mitochondria fraction were detected by western blotting. The values in the graphs **A**, **B** represent the mean ± SD of three independent experiments. *P < 0.01 compared to the control. ^#^*P* < 0.01 compared to ODN.

**Fig. 7:**
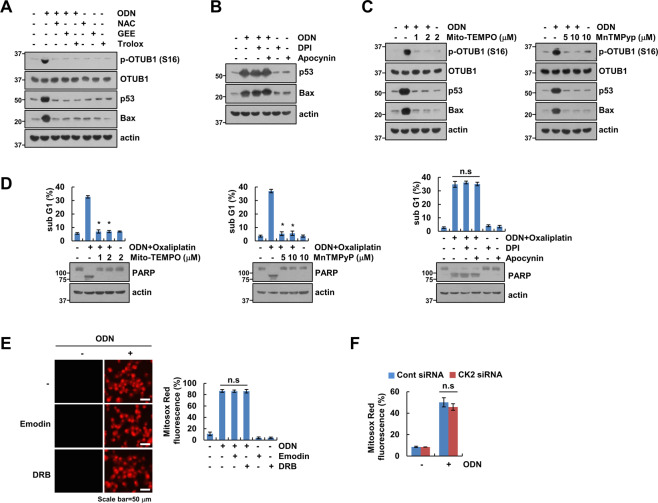
Mitochondrial ROS plays an important role in the combined effect of ODN and oxaliplatin, via the CK2/OTUB1/p53/Bax signaling pathway. **A–C** Caki-1 cells were pretreated for 30 min with an ROS scavenger (NAC, GEE, or Trolox) (**A**), a NOX inhibitor (apocynin) (**B**), or a mitochondrial ROS inhibitor (Mito-TEMPO or MnTMPyp) (**C**), and then treated with 2 μM ODN for 24 h. **D** Caki-1 cells were pretreated with a Mito-TEMPO, MnTMPyp, DPI or apocynin for 30 min, and then treated with a combination of 2 μM ODN and 25 μM oxaliplatin for 24 h. **E** Caki-1 cells were pretreated with CK2 inhibitors (emodin and DRB) for 30 min, and then treated with 2 μM ODN for 3 h. **F** Caki-1 cells were transfected with control siRNA or CK2 siRNA, and treated with 2 μM ODN for 3 h. Mitochondrial ROS production was analyzed by fluorescence microscope or flow cytometry using MitoSOX Red. Protein expression and apoptosis were measured by western blotting (**A–D**) and flow cytometry (**E**, **F**). The values in the graphs **D**–**F** represent the mean ± SD of three independent experiments. *P < 0.01 compared to the ODN-plus-oxaliplatin treatment.

ROS are important signaling molecules for anticancer drug-mediated cell death. Therefore, we investigated whether cathepsin K inhibition generates mitochondrial ROS. As shown in Fig. 6A, ODN and cathepsin K siRNA increased the intracellular ROS and mitochondrial ROS. To confirm the source of ROS generation, we used pHyPer-cyto, pHyPer-nuc, or pHyPer-dMito vector. As expected, pHyPer-dMito fluorescence was increased by ODN, but not pHyPer-nuc or pHyPer-nuc in Caki-1 cells (Fig. 6B). Blockers of mitochondrial ROS (Mito-TEMPO and MnTMPyP) significantly inhibited ODN-induced mitochondrial ROS (Fig. 6C). Since mitochondrial ROS is generated by inhibiting oxidative phosphorylation (OXPHOS) [16], ODN time-dependently downregulated expression levels of OXPHOS complex I (NDUF88) and complex II (SDHB), but not complex III (UQCRC2) and complex V (ATP5A) (Fig. 6D). Next, we investigated whether ODN-induced ROS production is involved in OTUB1 phosphorylation. ROS scavengers [N-acetyl-l-cysteine (NAC), glutathione monoethyl ester (GEE), and Trolox] completely blocked OTUB1 phosphorylation and inhibited p53 stabilization and Bax upregulation (Fig. 7A). To elucidate the source of ROS production related to ODN-mediated p53 stabilization, we employed mitochondrial ROS scavengers [Mito-TEMPO and Mn(III) tetrakis (1-methyl-4-pyridyl) porphyrin pentachloride (MnTMPyp)], and NADPH oxidase (NOX) inhibitors [diphenyleneiodonium (DPI) and apocynin]. The NOX inhibitors did not affect ODN-induced OTUB1 phosphorylation or the expression of p53 and Bax (Fig. 7B). However, the mitochondrial ROS scavengers (Mito-TEMPO and MnTMPyp) markedly inhibited OTUB1 phosphorylation and upregulation of p53 and Bax (Fig. 7C). Furthermore, ODN-plus-oxaliplatin-induced apoptosis was significantly inhibited by pretreatment with mitochondrial ROS scavengers, but not with NOX inhibitors (Fig. 7D). Next, we examined the relationship between mitochondrial ROS and CK2 activation. CK2 inhibition and knockdown did not affect ODN-induced mitochondrial ROS production (Fig. 7E, F). Therefore, CK2 might be downstream of the signaling pathway involved in mitochondrial ROS production in p53 WT Caki-1 cells.

### Cotreatment with ODN and oxaliplatin suppresses tumor growth in vivo

**Fig. 8:**
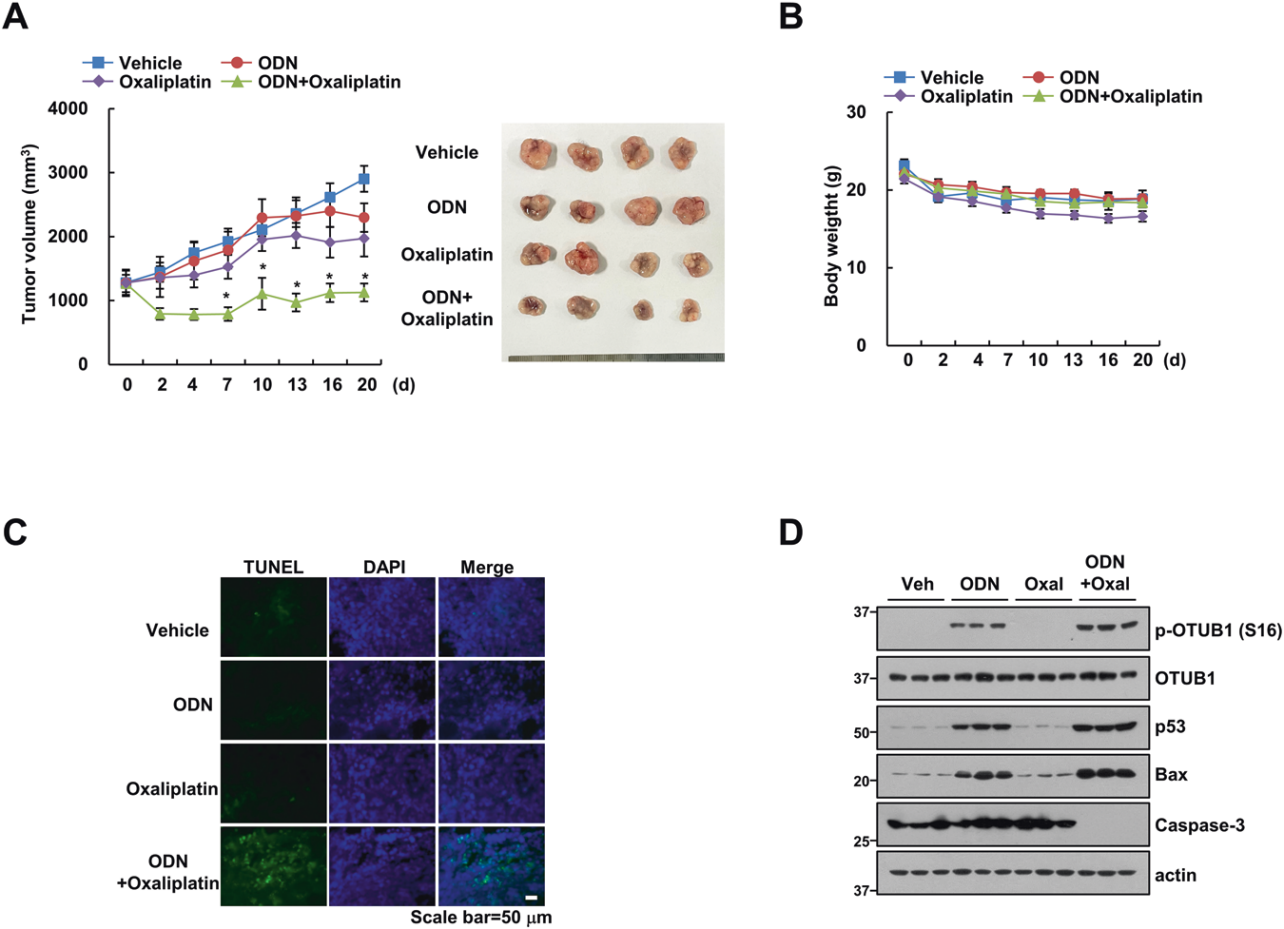
ODN reduces tumor growth in oxaliplatin-treated xenograft model. (**A–D**) Mice were treated with vehicle, 5 mg/kg ODN, 5 mg/kg oxaliplatin or a combination for 20 days. Tumor volume (**A**) and mice body weight (**B**) were measured. To check apoptosis, TUNEL assays were performed (**C**). Protein levels were detected by western blotting (**D**). *P < 0.01 compared to the vehicle. Values in the graphs **A, B** represent the mean ± SD of three independent experiments.

To verify the synergistic effect of ODN and oxaliplatin in an in vivo p53 WT HCT116 xenograft model, single (ODN or oxaliplatin) or combined treatment (ODN plus oxaliplatin) in tumor-bearing mice were applied. Single treatment showed a weak inhibitory effect on tumor volume, whereas combined treatment markedly reduced tumor volume (Fig. 8A). However, body weight did not change in combined treatment (Fig. 8B). Furthermore, TUNEL-positive signals were detected in combination treatment (Fig. 8C). We checked the related protein levels to ODN or/and oxaliplatin using in vivo samples. Similar to our in vitro results, ODN or ODN plus oxaliplatin increased Ser16 phosphorylation of OTUB1, p53, and Bax expression, whereas decreased caspase-3 expression (Fig. 8D). These data implied that combined treatment ODN and the anticancer drug has synergistic effects in the in vivo.

### Phosphorylation of OTUB1 correlates with p53 and Bax in human RCC patient tissues

**Fig. 9:**
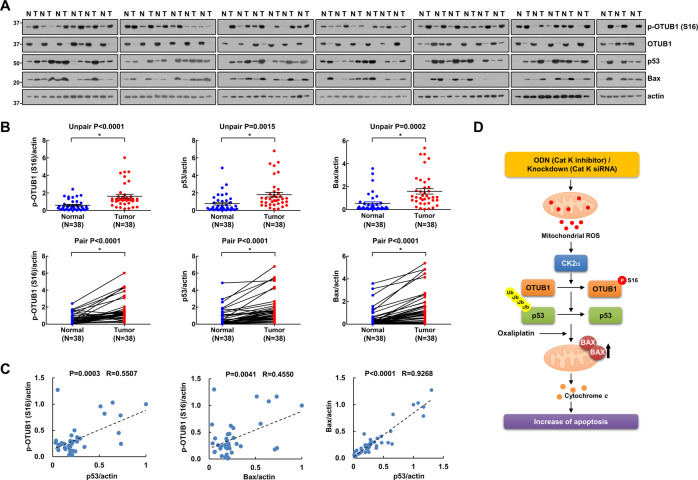
Expression levels of p-OTUB1(S16), p53, and Bax protein in renal tumor tissue. **A, B** Investigation of the protein expression in 38 unpaired (upper panel)/paired (lower panel) primary renal tumor tissues and corresponding normal adjacent ones. **C** Correlation analysis of protein expression of p-OTUB1/p53, p-OTUB1/Bax, or Bax/p53. **D** Scheme indicating the mechanism of oxaliplatin sensitization via cathepsin K inhibition.

We collected 38 specimens of human renal clear carcinoma (RCC) tissues and analyzed related proteins expression. The results revealed that OTUB1 Ser16 phosphorylation, p53, and Bax were upregulated in RCC tissues (Fig. 9A, B). The expression levels of three proteins were quantified in all samples, 84.2% (32/38) of OTUB1 Ser16 phosphorylation, 86.8% (33/38) of p53, and 81.6% (31/38) of Bax were significantly higher in tumor tissues compared to adjacent normal tissue, respectively (Fig. 9B). Moreover, Ser16 phosphorylation of OTUB1 has positive relationship with p53 and Bax (Fig. 9C). Collectively, our findings indicate that the mitochondrial ROS/CK2/OTUB1 phosphorylation/p53/Bax axis signaling pathway plays a critical role in ODN-plus-oxaliplatin-induced apoptosis.

## Discussion

In this study, we have demonstrated that cathepsin K inhibition enhanced oxaliplatin-induced apoptosis in p53 WT cancer cells. Both pharmacological inhibitions via ODN and genetic ablation of cathepsin K upregulated Bax, a proapoptotic protein. Cathepsin K inhibition-induced Bax upregulation was regulated by p53 stabilization, which was regulated by mitochondrial ROS-mediated CK2-dependent phosphorylation of OTUB1 at Ser16 in p53 WT cancer cells. Collectively, cathepsin K inhibition enhanced oxaliplatin-induced apoptosis by upregulating CK2-mediated OTUB1 phosphorylation, to induce p53 stabilization-mediated Bax expression (Fig. 9D).

We found that both specific inhibition and knockdown of cathepsin K increased Bax expression in multiple cancer cells lines (Figs. 2B and 3B). ODN-mediated Bax upregulation was modulated at the transcriptional level by p53 (Fig. 3C, D), but was not detected in p53-null cancer cell lines (Fig. 3C-F). Several studies have reported that Bax expression is regulated by p53-dependent transcriptional activation, occurring at two p53 half-sites plus an adjacent six base pairs (5′-GGGCGT-3′) [12, 17]. We also investigated whether ODN upregulates other p53-target proteins, such as p21, PUMA, Noxa, and DR5. However, ODN did not affect the expression of these proteins (Fig. S2). Based on a comparative analysis of p53 targets genes, it is known that Bax gene transcription is differentially regulated by p53 [18–20]. In our study, ODN stabilized p53 to induce Bax expression. Further, MDM2, a major E3 ligase of p53 [13, 21], was not involved in p53 stabilization (Fig. S1B-D). Therefore, we focused on the role of deubiquitinases in regulating p53 activity. OTUB1 had a critical role in p53 stabilization (Fig. 4A). OTUB1 performs both canonical and non-canonical deubiquitination [22]. Sun et al. have shown that OTUB1 directly suppresses MDM2-mediated p53 ubiquitination. Ectopic expression of OTUB1 stabilizes and activates p53, leading to apoptosis and inhibition of cell growth in a p53-dependent manner [23]. Wiener et al. reported that deleting the first 45 OTUB1 residues makes it unable to non-canonically inhibit E2 enzymes [24, 25]. Consistent with this observation, the N-terminal deletion mutant (Δ1-45) abolished ODN-induced p53 stabilization (Fig. 4D). Interestingly, ODN markedly increased phosphorylation of OTUB1 at Ser16 but did not increase OTUB1 expression (Fig. 4B). CK2 is known to regulate the function of OTUB1 via OTUB1 phosphorylation at Ser16 [15]. In our study, CK2 inhibition and knockdown also induced inhibition of OTUB1 phosphorylation and increased p53 stabilization (Fig. 5A, B). Therefore, CK2-induced OTUB1 phosphorylation is critical for p53 stabilization and nuclear localization in ODN-induced Bax upregulation.

ROS generation is tightly controlled, because an imbalance in redox reactions can impair cellular function. Cathepsin deficiency induces ROS production and mitochondrial dysfunction. Proteome analysis of cathepsin L-deficient myocardium revealed a significant reduction in the levels of respiratory chain components, compared with levels in the WT [26]. The use of cathepsin E-deficient macrophages revealed augmented ROS production and upregulation of oxidized peroxiredoxin-6, but reduced levels of the antioxidant glutathione [27]. Cathepsin S inhibition increases intracellular ROS levels by inducing mitochondrial dysfunction [28]. Recently, we reported that ODN induces mitochondrial fusion and mitochondrial ROS generation via raptor downregulation [6]. ODN-induced mitochondrial ROS are also tested by using pHyPer-dMito vector in this study (Fig. 6B). Also, ODN reduced OXPHOS complex I and II expression (Fig. 6D). Mitochondrial ROS blockers (Mito-TEMPO and MnTMPyp) inhibited ODN-induced phosphorylation of OTUB1 at Ser16 and upregulation of p53 and Bax (Fig. 7C). Mitochondrial ROS may be involved in regulating CK2 activation. Neither pharmacological inhibition nor genetic ablation of CK2 affected ODN-induced mitochondrial ROS production (Fig. 7E, F). Our findings suggest that mitochondrial ROS function as a signaling trigger upstream of CK2. However, further study is required to elucidate how mitochondrial ROS modulate CK2 activity.

In conclusion, our findings suggest that cathepsin K inhibition enhances oxaliplatin-induced apoptosis by upregulating Bax, a proapoptotic protein. Mitochondrial ROS production induced phosphorylation of OTUB1 at Ser16 via CK2 activation; phosphorylated OTUB1, in turn, increased p53 stabilization, resulting in Bax upregulation at the transcriptional level. Therefore, the combination of cathepsin K inhibition and anticancer drugs may provide a novel and effective strategy for cancer therapy.

## Materials and methods

### Cell culture and materials

All human cancer cells (Caki-1, ACHN, U87MG, MCF7, NCI-H1299, and SaOS-2) were obtained from the American Type Culture Collection (Manassas, VA). The normal mouse kidney (TCMK-1) cells were a gift from Dr. T. J. Lee (Yeungnam University, Korea), and human mesangial cells were purchased from Lonza (Basel, Switzerland). Cells were cultured in Dulbecco’s modified Eagle’s medium containing 10% fetal bovine serum (Welgene, Gyeongsan, Korea), 1% penicillin–streptomycin, and 100 µg/mL gentamycin (Thermo Fisher Scientific, Waltham, MA). More detailed information about the materials is described in Supplementary Table S1.

### Flow cytometry analysis

Harvested cells were resuspended in 100 µl phosphate-buffered saline (PBS) and fixed using 200 µl of 95% ethanol at 4 °C. After 1 h, the cells were washed with PBS, resuspended in 1.12% sodium citrate buffer (pH 8.4) containing 12.5 µg RNase, and incubated at 37 °C for 30 min. Then, 250 µl of propidium iodide solution (50 µg/ml) was added to the cells, followed by incubation at 37 °C for 30 min. The number of apoptotic cells was measured using a BD Accuri^TM^ C6 flow cytometer (BD Biosciences, San Jose, CA).

### Western blot analysis

Cells were lysed using RIPA buffer containing a protease inhibitor [29]. Cell lysates were collected by centrifugation at 13,000 × *g* for 15 min at 4 °C. Protein samples were separated using SDS-PAGE and transferred onto nitrocellulose membranes (GE Healthcare Life Science, Pittsburgh, PA). Protein bands were detected using an enhanced chemiluminescence kit (EMD Millipore, Darmstadt, Germany).

### DNA fragmentation and Asp-Glu-Val-Asp-ase (DEVDase) activity assay

The amount of fragmented DNA was detected using the Cell Death Detection ELISA Plus kit (Roche, Basel, Switzerland) [6]. For the analysis of caspase-3 activation, 20 µg of cell lysates were incubated with an acetyl-Asp-Glu-Val-Asp p-nitroanilide (Ac-DEVD-pNA) for 2 h at 37 °C and measured at 405 nm absorbance using a spectrophotometer [6].

### Detection of mitochondrial membrane potential and cytochrome *c* release

Mitochondrial membrane potential (MMP) was analyzed using a 5 µM rhodamine 123 dye (Molecular Probes Inc., Eugene, OR), and fluorescence was measured by BD Accuri^TM^C6 flow cytometer (BD Biosciences). For the analysis of cytochrome *c* release [30], cell lysates were centrifuged at 13,000 × *g* for 15 min at 4 °C. The supernatants (cytosolic extract) and the pellets (mitochondrial extract) were collected. The amount of cytochrome *c* released into the cytoplasm was analyzed using western blotting.

### Transfection

Cells were transiently transfected with siRNA using Lipofectamine RNAiMAX (Thermo Fisher Scientific) or with plasmids using Lipofector p-MAX (AptaBio, Korea).

### Reverse transcription PCR

Reverse transcription PCR (RT-PCR) and quantitative PCR were performed as previously described [31]. The primer sequences used are described in Supplementary Table 1.

### Promoter activity measurement

Cells were transfected with the Bax/−600 luciferase promoter, using Lipofector p-MAX (AptaBio, Korea), and were treated with ODN for 12 h. The lysates were then incubated with the luciferase substrate, luciferin (Promega, Madison, WI).

### Bax-activation analysis

Cells were harvested and fixed by adding 4 % paraformaldehyde for 30 min. Cells were incubated with the Bax (6A7) antibody in PBS / 1% FCS / 0.1% saponin for 1 h at 4 °C. The cells were then washed using PBS / 1% FCS, and incubated with secondary antibody in PBS / 1% FCS / 0.1% saponin for 1 h at 4 °C. Bax activation and oligomerization were the same as described previously [30].

### Deubiquitination assay

This assay was performed using a tagged-ubiquitin plasmid and pretreatment with MG132, as previously reported [32]. Briefly, the cells were cotransfected with HA-tagged ubiquitin (HA-Ub) and Flag-OTUB1 wild-type (WT), Flag-OTUB1 ∆1-45, and Flag-OTUB1 S16A plasmids, and then treated with MG132 for 12 h. Immunoprecipitation was performed using an anti-p53 antibody. Ubiquitination of endogenous p53 was identified using HRP-conjugated anti-Ub under denaturation conditions.

### ROS production assay

After treatment, the cells were incubated with H_2_DCF-DA or MitoSOX Red (Thermo Fisher Scientific, Waltham, MA) for 10 min at 37 °C, then harvested and resuspended in PBS. To examine source of ROS, we transfected using pHyPer-cyto, pHyPer-nuc, or pHyPer-dMito vector (Evrogen, Moscow, Russia) in the cells. ROS production through fluorescence was measured using BD Accuri™ C6 flow cytometer (BD Biosciences) or fluorescence microscope.

### Xenograft model

Male BALB (bagg and albino)/c-nude mice were purchased from the JA BIO, Inc. (Suwon, Korea), and mice were maintained in a pathogen-free conditioned room. The IRB Keimyung University Ethics Committee (KM-2020-03R2) approved our study protocol. HCT116 cells were subcutaneously injected on each flank of mice. After 3 weeks, mice were randomly divided into four groups and injected intraperitoneal (i.p.) of 5 mg/kg ODN (in 2% DMSO/PBS) or 5 mg/kg oxaliplatin (PBS) three times a week. The tumor size (length × width^2^)/2 was measured every time using a Vernier’s caliper (Mitutoyo Co., Tokyo, Japan). Apoptosis was detected ApopTag Fluorescein in situ Apoptosis Detection Kit (Millipore).

### Patient specimens

A total of 38 patients diagnosed with RCC were included in this retrospective study. RCC tissues were collected from patients undergoing surgery in Keimyung University Dongsan Medical Center (Daegu, Korea). Tissue samples were immediately frozen in liquid nitrogen and stored at −196 °C until Western blotting analysis. Tissue samples were provided by the Biobank of Keimyung University Dongsan Hospital Biobank (IRB-2019-11-040).

### Statistical analysis

Statistical analyses were performed using Statistical Package for Social Science (SPSS, Version 26.0; IBM SPSS, Armonk, NY, USA). All experiments were repeated three times or more. The data are presented as means. The data were analyzed using a one-way ANOVA and post-hoc comparisons (Student-Newman–Keuls). We determined the sample size based on the smallest effect we wished to measure. The association between the relative expression levels of p-OTUB1 (S16), p53, and Bax has assessed the Pearson’s correlation coefficients for continuous variables. *R* is Pearson’s correlation coefficient value. *P* < 0.05 was considered statistically significant.

## Supplementary information

Supplementary information
