# Supplementary material for: Inhibition of cathepsin K sensitizes oxaliplatin-induced apoptotic cell death by Bax upregulation through OTUB1-mediated p53 stabilization in vitro and in vivo
Source: Oncogene. 2021 Nov 16;41(4):550–9. doi: 10.1038/s41388-021-02088-7 (PMC8782718; doi:10.1038/s41388-021-02088-7)
Supplement: Supplementary file 1 — Supplementary information [file 41388_2021_2088_MOESM1_ESM.docx]

**Supplemental material**

**Supplemental Figure S1**

**Supplemental Figure S2**

**Supplemental Table S1**

**Supplemental Methods**

**
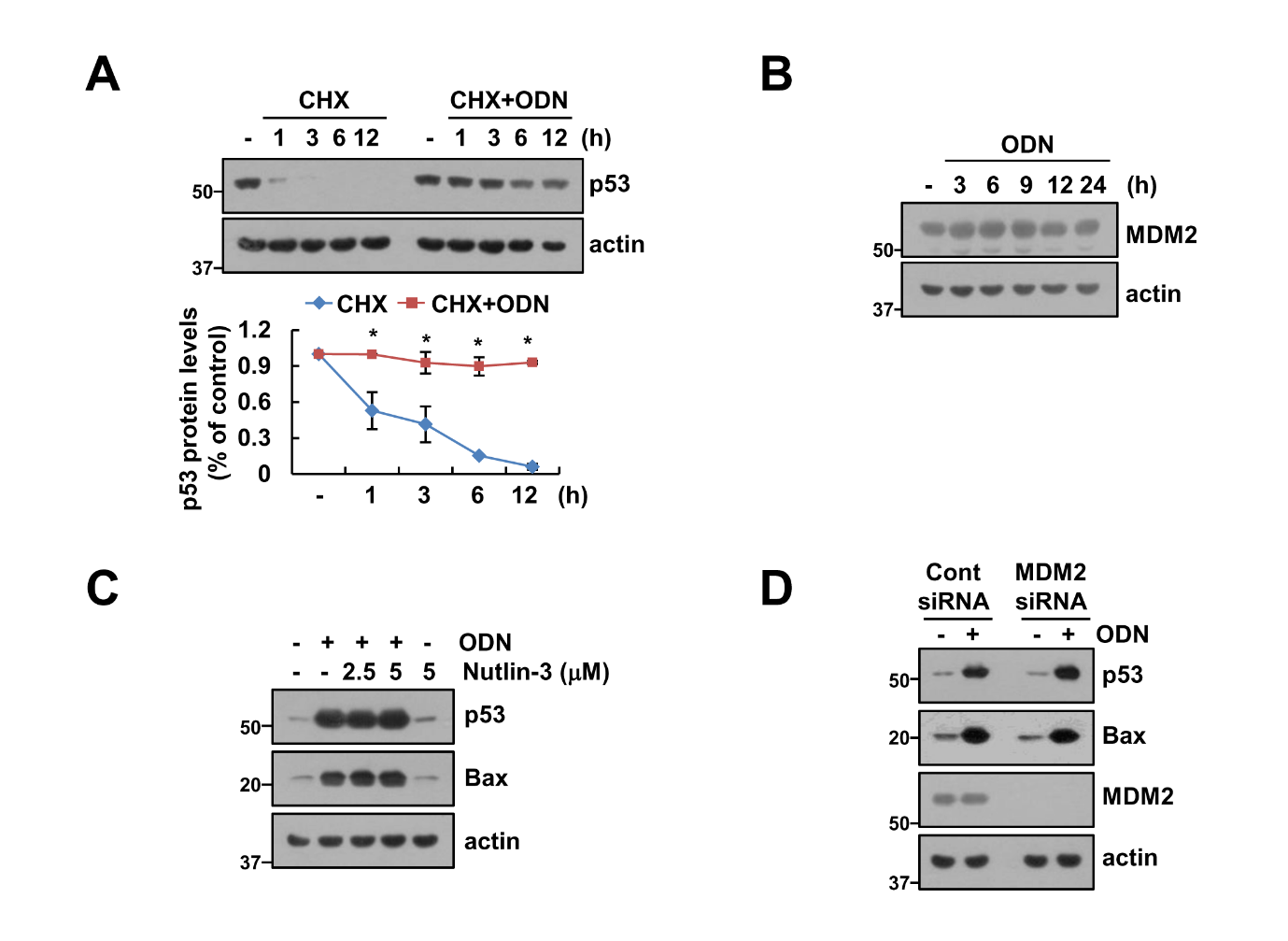
**

**Fig. S1** **ODN stabilizes p53 protein.** (**A**) Caki-1 cells were treated with 20 μg/mL CHX in the presence or absence of 2 μM ODN for the indicated times. (**B**) Caki-1 cells were treated with 2 μM ODN for the indicated times. (**C**) Caki-1 cells were pretreated with Nutlin-3 for 30 min and then treated with 2 μM ODN for 24 h. (**D**) Caki-1 cells were transfected with control siRNA or MDM2 siRNA, and treated with 2 μM ODN for 24 h. Protein expression was measured using Western blotting (**A-D**). The values in the graphs (**A**) represent the mean ± SD of three independent experiments. * *P* < 0.01 compared to the cells treated only with CHX.

**
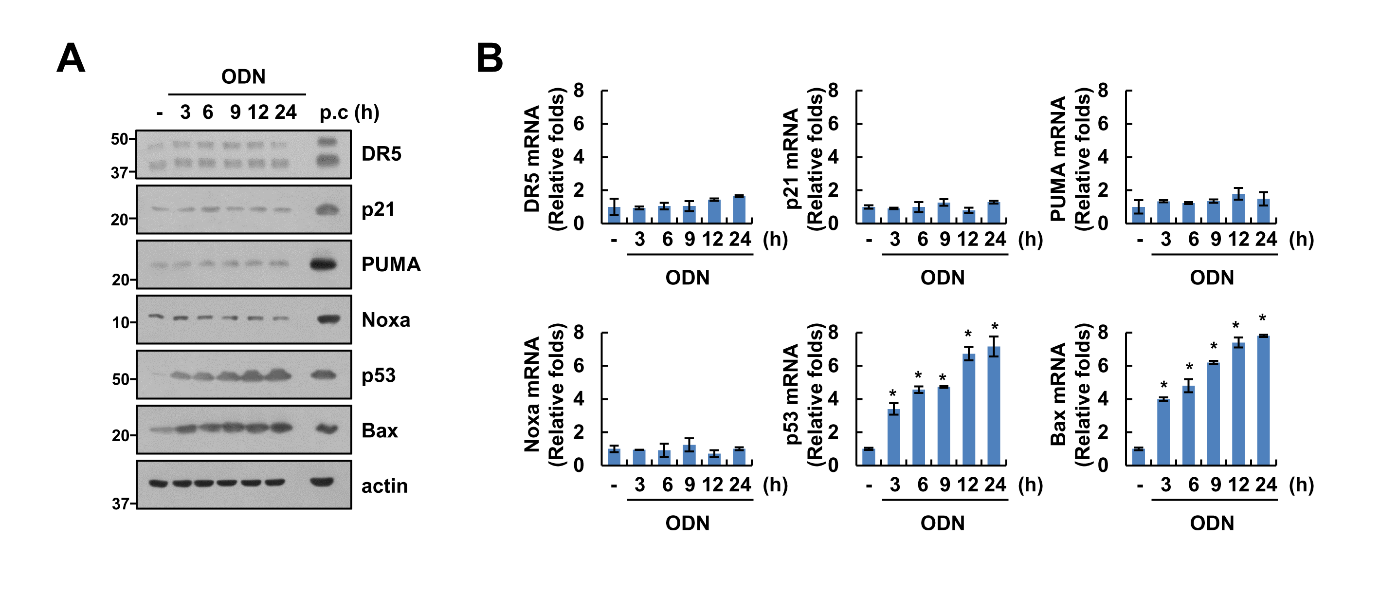
**

**Fig. S2** **ODN induces up-regulation of p53 and Bax expression.** (**A-B**) Caki-1 cells were treated with 2 μM ODN for the indicated time periods (p.c; 3 μg/ml etoposide for 24 h. Protein and mRNA expression were measured by western blotting (**A**) and real-time PCR (**B**). The values in the graphs (**B**) represent the mean ± SD of three independent experiments. * *P* < 0.01 compared to the control.

**Table S1. Reagent and Antibody**

| Reagent or Antibody | | Source | Identifier | |  |
| --- | --- | --- | --- | --- | --- |
| Apocynin | Sigma-Aldrich | | | Cat#178385 | |
| Cycloheximide (CHX) | Sigma-Aldrich | | | Cat# 01810 | |
| DPI | Sigma-Aldrich | | | Cat#D2926 | |
| DRB | Sigma-Aldrich | | | Cat#D1916 | |
| Emodin | Sigma-Aldrich | | | Cat#E7881 | |
| Etoposide | Sigma-Aldrich | | | Cat# E1383 | |
| GEE | Sigma-Aldrich | | | Cat#G6503 | |
| MG132 | Sigma-Aldrich | | | Cat# M8699 | |
| Mito-TEMPO | Sigma-Aldrich | | | Cat# SML 0737 | |
| MnTMPyP | Sigma-Aldrich | | | Cat#475872 | |
| NAC | Sigma-Aldrich | | | Cat#A7250 | |
| Nutlin-3 | Sigma-Aldrich | | | Cat#N6287 | |
| Odanacatib | Cayman chemical | | | Cat# 21466 | |
| Oxaliplatin | Sigma-Aldrich | | | Cat# O9512 | |
| Trolox | Sigma-Aldrich | | | Cat#648471 | |
| z-VAD-fmk | R&D Systems | | | Cat# FMK001 | |
| Bax | BD Biosciences | | | Cat#554104 | |
| Bax(6A7) | BD Biosciences | | | Cat#556467 | |
| Bcl-2 | Santa Cruz Biotechnology | | | Cat# sc-7382 | |
| Bcl-xL | Cell Signaling Technology | | | Cat# 2764 | |
| c-FLIP | Enzo Life Sciences | | | Cat# ALX-804-961-0100 | |
| Cathepsin K | Abcam | | | Cat# ab207086 | |
| cIAP1 | Abcam | | | Cat#ab15425 | |
| cIAP2 | Santa Cruz Biotechnology | | | Cat# sc-7944 | |
| CK2α | Santa Cruz Biotechnology | | | Cat# 12738 | |
| Cleaved-caspase3 | Cell Signaling Technology | | | Cat# 9661 | |
| Cytochrome *c* | BD Biosciences | | | Cat#556432 | |
| DR4 | Abcam | | | Cat# ab8414 | |
| DR5 | Cell Signaling Technology | | | Cat# 8074 | |
| Flag | Sigma-Aldrich | | | Cat# F1804 | |
| Mcl-1 | Santa Cruz Biotechnology | | | Cat# sc-12756 | |
| MDM2 | Santa Cruz Biotechnology | | | Cat#sc-813 | |
| MnSOD | Sigma-Aldrich | | | Cat#06-984 | |
| Noxa | Sigma-Aldrich | | | Cat#OP180 | |
| OTUB1 | Santa Cruz Biotechnology | | | Cat#sc-130458 | |
| p-OTUB1 (ser16) | Affinity Biosciences | | | Cat#AF3558 | |
| OXPHOS | Abcam | | | Cat#AB110413 | |
| p21 | Santa Cruz Biotechnology | | | Cat# sc-6246 | |
| p53 | Santa Cruz Biotechnology | | | Cat#sc-126 | |
| PARP | Cell Signaling Technology | | | Cat# 9542 | |
| Pro-caspase3 | Enzo Life Sciences | | | Cat# ADI-AAP-113 | |
| PUMA | Cell Signaling Technology | | | Cat#12450 | |
| Ref1 | Santa Cruz Biotechnology | | | Cat# sc-5572 | |
| Survivin | R&D Systems | | | Cat# AF886 | |
| TRIM2 | Sigma-Aldrich | | | Cat# SAB4200206 | |
| Ub | Santa Cruz Biotechnology | | | Cat# sc-8017 | |
| Ub-HRP | Enzo Life Sciences | | | Cat# BML-PW0150-0100 | |
| XIAP | BD Biosciences | | | Cat# 610762 | |
| Bax siRNA (h) | Santa Cruz Biotechnology | | | Cat# sc-29212 | |
| Cathepsin K siRNA (h) | Santa Cruz Biotechnology | | | Cat# sc-29936 | |
| CK2α siRNA (h) | Santa Cruz Biotechnology | | | Cat# sc-29918 | |
| GFP (control) siRNA | Bioneer | | | 5`- GUU CAG CGU GUC CGG CGA G -3` | |
| MDM2 siRNA (h) | Santa Cruz Biotechnology | | | Cat# sc-72124 | |
| OTUB1 siRNA | Bioneer | | | 5`-GAC AAC AUC UAU CAA CAG A -3` | |
| p53 siRNA (h) | Santa Cruz Biotechnology | | | Cat# sc-29435 | |
| **Oligonucleotides** |  | | |  | |
| H-actin-F | Bioneer | | | 5`-GGC ATC GTC ACC AAC TGG GAC -3` | |
| H-actin-R | Bioneer | | | 5`-CGA TTT CCC GCT CGG CCG TGG -3` | |
| H-Bax-F | Macrogen | | | 5`-ACC AAG AAG CTG AGC GAG TGT C-3` | |
| H-Bax-R | Macrogen | | | 5`-TGT CCA AGC CCA TGA TGG TTC-3` | |
| H-p53-F | Macrogen | | | 5`-GAA GAC CCA GGT CCA GAT GA-3` | |
| H-p53-R | Macrogen | | | 5`-CTC CGT CAT GTG CTG TGA CT-3` | |
| H-actin-F (qPCR) | Bioneer | | | 5`-CTA CAA TGA GCT GCG TGT G-3` | |
| H-actin-R (qPCR) | Bioneer | | | 5`-TGG GGT GTT GAA GGT CTC-3` | |
| H-Bax-F (qPCR) | Bioneer | | | 5`-CCC GAG AGG TCT TTT TCC GAG-3` | |
| H-Bax-R (qPCR) | Bioneer | | | 5`-CCA GCC CAT GAT GGT TCT GAT-3` | |
| H-DR5-F (qPCR) | Bioneer | | | 5`-AGA CCC TTG TGC TCG TTG TC-3` | |
| H-DR5-R (qPCR) | Bioneer | | | 5`-TTG TTG GGT GAT CAG AGC AG-3` | |
| H-Noxa-F (qPCR) | Bioneer | | | 5`-GCA GAG CTG GAA GTC GAG TG-3` | |
| H-Noxa-R (qPCR) | Bioneer | | | 5`-GAG CAG AAG AGT TTG GAT ATC AG-3` | |
| H-p21-F (qPCR) | Bioneer | | | 5`-CGC TCT ACA TCT TCT GCC TT-3` | |
| H-p21-R (qPCR) | Bioneer | | | 5`-AAC CTC TCA TTC AAC CGC CT-3` | |
| H-p53-F (qPCR) | Bioneer | | | 5`-TCT GAG TCA GGA AAC ATT TCC- 3` | |
| H-p53-R (qPCR) | Bioneer | | | 5`-CTG GGA AGG GAC AGA AGA TGA-3` | |
| H-PUMA-F (qPCR) | Bioneer | | | 5`-GAC GAC CTC AAC GCA CAG TA -3` | |
| H-PUMA-R (qPCR) | Bioneer | | | 5`-AGG AGT CCC ATG ATG AGA TTG T-3` | |
| **Plasmids** |  | | |  | |
| pcDNA3.1(+) | | Thermo Fisher Scientific | Cat#V79020 | |  |
| pCMV-Neo-Bam p53 WT | | Addgene | Cat# 16434 | |  |
| HA-Ubiquitin | | Addgene | Cat# 18712 | |  |
| pcDNA3-Flag-HA-OTUB1 WT | | Were a gift from Ph.D.E.W.Lee  (Korea Research Institute of Bioscience  & Biotechnology) | | |  |
| pcDNA3-Flag-HA-OTUB1 ∆1-45 | |  |  |  |  |
| pcDNA3-Flag-HA-OTUB1 S16A | |  |  |  |  |

**Supplemental Methods**

**Quantitative PCR (qPCR)**

The quantitative PCR (qPCR) was measured using SYBR Fast qPCR Mixture and performed on Thermal Cycler Dice® Real Time System III (Takara Bio Inc., Shiga, Japan). The information of used primer in this study is described in Table S1.
